# Phenotypic screening and molecular characterization of carbapenemase-producing Gram-negative bacilli recovered from febrile neutropenic pediatric cancer patients in Egypt

**DOI:** 10.1371/journal.pone.0202119

**Published:** 2018-08-29

**Authors:** Noha A. Kamel, Wafaa N. El-tayeb, Mona R. El-Ansary, Mohamed T. Mansour, Khaled M. Aboshanab

**Affiliations:** 1 Department of Microbiology, Faculty of Pharmacy, Misr International University (MIU), Cairo, Egypt; 2 Department of Biochemistry, Modern University for Technology and Information, Cairo, Egypt; 3 Department of Virology and Immunology, 57357 Children’s Cancer Hospital, Cairo University, Cairo, Egypt; 4 Department of Microbiology & Immunology, Faculty of Pharmacy, Ain Shams University, Cairo, Egypt; Ain Shams University, EGYPT

## Abstract

**Aim:**

Infections with carbapenem-resistant Gram-negative bacteria (GNB) are among the most frequent complications in the immunocompromised cancer patients because of their considerable morbidity and mortality. Therefore, the aim of the current study was to characterize the prevalence of carbapenemase-producing GNB recovered from febrile neutropenic pediatric cancer patients in Egypt.

**Methods:**

Standard methods were used for identification, sensitivity testing (Kirby-Bauer and broth microdilution method according to CLSI guidelines). Standard methods were applied for both phenotypic and genotypic detection of the carbapenemase-producing GNB.

**Results:**

A total of 185 GNB were recovered from different clinical specimens, *Escherichia (E*.*) coli* (86; 46.48%), followed by *Klebsiella* spp. (71; 38.37%), *Acinetobacter (A*.*) baumannii* (7; 3.78%) and others including *Pseudomonas* spp., *Enterobacter (Ent*.*) cloacae* and *Proteus* spp. (21; 11.35%). It is a matter of concern that 116 out of 171 enterobacterial isolates (94.15%) showed resistance to three or more antimicrobial classes and were considered multidrug resistant. Additionally, the rate of carbapenem-resistance displayed a worrisome trend as 113 out of 171 enterobacterial isolates (66.08%) and 12 out of 14 non fermenting bacilli (85.71%) showed resistance pattern to at least one of the tested carbapenems. After performing a series of phenotypic tests for initial screening of potential carbapenemase producers, molecular characterization to the 29 extracted plasmids were subjected to PCR (using 5 common carbapenemase primers). The results revealed that *bla*_OXA-48_ was the most prevalent 17 (58.62%), followed by *bla*_NDM_ 8(27.58%), then *bla*_VIM_ 3 (10.3%) and *bla*_KPC_ 2 (6.89%).

**Conclusion:**

These results are an alarming threat to public health that calls for urgent application of antimicrobial stewardship programs along with routine surveillance for controlling outbreaks.

## Introduction

Immunodeficiency is a common problem among cancer patients that results from the underlying disease itself, chemotherapy that induce neutropenia, and/or radiation therapy[1]. Classically, neutropenic cancer patients are infected with *E*. *coli* and *Klebsiella* spp. that are endogenously acquired through gastrointestinal tract after initiation of cytotoxic chemotherapy. Recently, there has been a dramatic increase in the proportion of bloodstream infections among cancer patients caused by non fermenting bacilli including *Pseudomonas aeruginosa*, *A*. *baumannii* and *St*. *maltophilia* along with the evolution of multiple drug resistant phenotype (MDR) [2,3].Of particular concern is the emergence of carbapenem-resistant *Enterobacteriaceae* (CRE), MDR *Pseudomonas* and *Acinetobacter* species[4].

Resistance to carbapenem could be through decrease in outer membrane permeability accompanied with hyperproduction of AmpC β-lactamases, production of extended-spectrum beta-lactamases (ESBLs) or expression of carbapenemase enzymes. However, the ability to produce class A, B and D carbapenemases remain by far the most clinically important carbapenem-resistance mechanism[5–8]. Currently, there are 5 main carbapenemase enzymes that are widely involved in nosocomial outbreak including, *Klebsiella pneumoniae* carbapenemase (KPC), Verona integron encoded metallo-β-lactamase (VIM), New Delhi metallo-β-lactamase (NDM) and for activity on imipenem(IMP) and oxacillinases due to their ability to hydrolyze oxacillin at a relatively high rate (OXA-48)[9]. Owing to high mortality rates, resistance to commonly used antibiotics that limits treatment options and the great potential for widespread dissemination, rapid detection of carbapenemase-producers (CP) is of vital importance for controlling this alarming public health issue[10]. Accordingly, the present study aimed to determine the prevalence of CP-GNB among children with malignancy by using phenotypic screening test in addition to molecular assays.

## Methods

### Collection and examination of clinical isolates

A total of 185GNB clinical isolates were collected from bacteriology unit of 57357 children’s cancer hospital, Cairo, Egypt during the period of October 2014 to December 2016. The study was approved by the hospital Ethics Committee and faculty of Pharmacy ethical committee Nr. 72 where both informed and written consents were obtained from parents of patients after explaining the study purpose. The isolates were recovered from various clinical specimens including blood (n = 146), urine (n = 4), stool (n = 11), wound (n = 12)and others including catheter tip and sputum (12)from 185 cancer pediatric patients (averaged age 3.5 years) patients having absolute neutrophils count (ANC) <500/ mm3 and oral temperature >38°C over at least 1 hour. The blood specimens were injected into Bactec® (Becton Dickinsion, USA) culture vials and incubated in the Bactec 9050® (Becton Dickinsion, USA) incubator. Positive culture specimens were subcultured on blood agar, chocolate agar and MacConkey agar (Oxoid, England) plates. After overnight incubation at 37°C, the colonial appearance and distinguishing characters were recorded. Isolates were identified macroscopically, microscopically and standard biochemical tests according to Bergey’s manual of determinative bacteriology were performed[11].

### Antimicrobial susceptibility testing by disk diffusion and determination of minimum inhibitory concentration

The antibiotic susceptibility testing was determined by Kirby-Baurer method and a panel of 18 antibiotics disks including amoxicillin/clavulanic acid (20 μg/10 μg), amikacin (30 μg), aztreonam (30 μg), cefepime (30 μg), cefotaxime (30 μg), ceftriaxone (30 μg), ciprofloxacin (5 μg), colistin (10 μg), doripenem (10 μg), ertapenem (10 μg), fosfomycin (50 μg), gentamicin (10 μg), imipenem (10 μg), polymyxin B (300 U), rifampicin (5 μg), sulphamethoxazole/trimethoprim (25 μg), tetracycline (30 μg) and tigecycline (15 μg) were tested. Kirby-Bauer test was done once for each tested isolate and the diameter of inhibition zone was measured. Isolates that showed resistance to at least one agent in three or more antimicrobial category were considered multidrug resistant (MDR) [12]. Isolates that showed resistance pattern to any of the carbapenems tested (by Kirby-Bauer) were considered carbapenem-resistant and were subsequently chosen to determine their minimum inhibitory concentration (MIC) against ertapenem and imipenem by broth microdilution method. Broth microdilution test was done in triplicate and isolates that showed MIC of≥4 μg/ml for meropenem or ≥2 μg/ml for imipenem were considered carbapenem-resistant isolates with high potential for carbapenemase production[13].The reference strain *E*. *coli* ATCC^®^ 25922^TM^ was used as a quality (susceptible) control.

### Phenotypic detection of carbapenemase producers

#### Modified Hodge test

Modified Hodge test (MHT) was carried using ertapenem and meropenem as described by CLSI, 2015. Mueller-Hinton agar plates were inoculated by an overnight culture of *E*. *coli* ATCC^®^ 25922^TM^adjusted to one tenth turbidity of 0.5 McFarland. The plates were left for 15 minutes to dry and then ertapenem or meropenem discs were placed at the center of the plate. Overnight cultures of the tested isolates (3–5 colonies) were streaked from the edge of disc to the periphery of the plates and the plates were overnight incubated at temperature 37°C. Carbapenemase-producer isolates were indicated by enhanced growth of indicator *E*. *coli* expressed as clover leaf like indentation, while absence of growth of *E*. *coli* along the streak of tested isolates indicates non carbapenemase-producing isolate[14].Modified Hodge test was done in duplicate as an initial screening test for determination of carbapenemase-production among carbapenem-resistant isolates. Isolates that showed negative results were re-tested again for confirmation.

#### Modified carbapenem inactivation method

Recently in 2017, the CLSI had recommended modified carbapenem inactivation method (mCIM) for detection of carbapenemase producers (CPs) using readily available laboratory reagents. Briefly, a meropenem disk was immersed and incubated for at least 4 hours in a bacterial suspension of tested strain. Thereafter the disk was transferred to a plate inoculated with *E*. *coli* ATCC^®^ 25922^TM^and the plate was overnight incubated. Tested isolates that showed a zone of inhibition between 6-15mm or colonies were present within 16-18mm were considered CPs, while isolates that showed a zone of inhibition greater than or equal to 19mm were not considered CPs[15]. Modified carbapenem inactivation method was done in duplicate on promising CPs isolates after plasmid extraction.

#### Blue carba test

For rapid detection of CP directly from bacterial culture blue carba biochemical test was used. A test solution of 0.04% bromothymol blue adjusted to pH = 6, 0.1 mmol/liter ZnSo_4_ and 3mg/ml of imipenem (immediately added before using test) with a final pH adjusted to 7 was prepared. A negative control of 0.04% bromothymol blue adjusted to pH = 7 was prepared to check influence of bacterial components on pH of solution. A loopful (about 5μl) of pure bacterial culture were added to both test and negative control solution in a 96 well microtiter plate. The plates were incubated at 37°C for 2 hours and carbapenemase activity was determined when test and negative control solution were respectively, yellow versus blue, yellow versus green and green versus blue. Non CPs failed to change color of indicator and both solution remained either blue or green[16–18]. Blue carba test was done in duplicate on the promising CPs isolates after plasmid extraction.

#### Combined disk test

For determination of type of carbapenemase involved in resistance, combined disk test was used. An overnight culture of tested isolate adjusted to 0.5 McFarland standard was streaked over the surface of Mueller-Hinton agar. Meropenem, meropenem/Boronic acid (for class A carbapenemase) and imipenem, imipenem/ EDTA (for class B carbapenemase) were placed on the surface of the plate using sterile forceps. Enhancement of inhibition zone (≥ 5mm) of carbapenem disk with inhibitor compared to carbapenem alone after overnight incubation was considered a positive confirmatory test for carbapenemase production[19]. Combined disk test was done on CPs isolates in duplicate to confirm the presence of carbapenemase class A and class B.

### Plasmid extraction

For the detection of plasmid mediated carbapenemase genes among our isolates, the phenotypically confirmed producers by the previously mentioned tests were subjected to plasmid extraction by DNA-spin^TM^(Intron Biotechnology,Korea). An overnight culture of the tested isolates was grown on Luria Bertani broth containing 125μg/ml ertapenem as a selective pressure to enhance plasmid recovery. The extracted DNA was analyzed using 0.8% agarose gel electrophoresis[20]

### DNA amplification by PCR

The extracted plasmids of phenotypically confirmed CPs were used as a template for PCR. The following basic cycles were used for PCR amplification; initial denaturation of the target DNA sequence at 95°C for 2 minutes (1 cycle), denaturation at 95°C for 30 seconds (30 cycles), annealing of both forward and reverse primers of (NDM/OXA-48 and VIM) at 54°C for 45 seconds, while annealing of both forward and reverse primers of (KPC and IMP) at 51°C for 45 seconds for 30 cycles. extension step of the primers at 72°C for 90 seconds for 30 cycles. a post extension step was conducted for 5 minutes at 72°C (1 cycle).A multiplex PCR test was used to determine the five most predominantcarbapenemase genes as mentioned in Table 1.

**Table 1:** Primer sequences and expected sizes of PCR products of carbapenemase genes.

Carbapenemase genes	Forward primer (5’-3’)	Reverse primer (5’-3’)	Expected PCR product size	Tm	reference
*bla*_KPC_	TGTCACTGTATCGCCGTC	TATTTTTCCGAGATGGGTGAC	331 bp	56	[21]
*bla*_IMP_	CTACCGCAGCAGAGTCTTTG	AACCAGTTTTGCCTTACCAT	587 bp	56	[21]
*bla*_VIM_	TCTACATGACCGCGTCTGTC	TGTGCTTTGACAACGTTCGC	748 bp	59	[21]
*bla*_NDM_	GGTTTGGCGATCTGGTTTTC	CGGAATGGCTCATCACGATC	621 bp	57	[22]
*bla*_OXA-48_	GCGTGGTTAAGGATGAACAC	CATCAAGTTCAACCCAACCG	438 bp	57	[23]

## Results

### Identification of clinical isolates

Out of 185 GNB recovered from the different clinical specimens during the course of the study, 171 were enterobacterial isolates and 14 were non fermentive bacilli. The enterobacterial isolates included 86 *E*. *coli*, 71 *Klebsiella* spp., 5 *Ent*. *cloacae*, 5 *Proteus* spp., 3 *Salmonella* spp. and 1 *Serratia marcescens* while non fermentive included 7 *A*. *baumannii*., 4 *Pseudomonas* spp., and 3 *St*. *maltophilia*. The isolates were collected on routine workdays without any specific exclusion criteria. For confirmation of the identified isolates, our data were compared with data records of the hospital.

### Antibiogram analysis of recovered GNB

Table 2 revealed that resistance profile of *E*. *coli* exceeded 50%; for all β- lactam group except for imipenem (46%), doripenem (48.2%), gentamicin, ciprofloxacin, sulfamethoxazole/trimethoprim, tetracycline, and fosfomycin. A remarkable reduced resistance profile was recorded for polymyxins group, tigecycline, and amikacin. The resistance profile of *Klebsiella* spp. exceeded 50%; for all β- lactam group, aminoglycosides class,ciprofloxacin, sulfamethoxazole/trimethoprim, tetracycline, and fosfomycin.A remarkable reduced resistance profile was recorded for polymyxins group and tigecycline. The resistance profile of *A*. *baumannii* was equal to or greater than 50%; for all β- lactam group, aminoglycosides class,ciprofloxacin, sulfamethoxazole/trimethoprim, tetracycline, and fosfomycin. A reduced resistance profile was recorded for polymyxins group, tigecycline and rifamycin. The resistance profile of *Ent*. *cloacae* exceeded 50%; for all β- lactam group except for aztreonam, imipenem and doripenem that reached 40% each, sulfamethoxazole/trimethoprim, tetracycline, fosfomycin and rifamycin. The resistant profile reached 40% to amikacin, ciprofloxacin and tigecycline. None of tested isolate was resistant to colistin, while 20% were resistant topolymyxin B. The resistance profile of *St*. *maltophilia*exceeded 50%; for all β- lactam group, ciprofloxacin, fosfomycin and rifamycin.The resistance profile reached 33.33% to amikacin, gentamicin, sulfamethoxazole/trimethoprim, tetracycline, ciprofloxacin and tigecycline. None of tested isolate was resistant to polymyxins. The resistance profile of *Pseudomonas* spp. was equal to or greater than 50%; for all β- lactam group except cefepime and doripenem. All the tested isolates were resistant to sulfamethoxazole/trimethoprim, tigecycline, fosfomycin and rifamycin. None of tested isolates were resistant to polymyxins. The resistance pattern reached 25% for aminoglycoside class.

**Table 2:** Antimicrobial resistance patterns of the recovered GNB.

Antimicrobialclass	Antimicrobial agent	*E*. *coli* ^*A*^	*Klebsiella* spp.^B^	*A*. *baumannii*^C^	*Ent*. *cloacae*	*St*. *maltophilia*	*Pseudomonas* spp.
β-lactam group	Amoxicillin/ clavulanic acid (AMC)	90.58%	95.23%	80%	100%	100%	100%
	Aztreonam (ATM)	85.88%	93.65%	60%	40	66.66%	75%
	Cefotaxime (CTX)	100%	98.59%	71.42%	100%	100%	100%
	Ceftazidime (CAZ)	94.18%	92.18%	83.33%	80%	66.66%	50%
	Ceftriaxone (CRO)	100%	96.82%	100%	100%	100%	100%
	Cefepime (FEP)	98.82%	93.65%	50%	80%	100%	25%
	Doripenem (DOR)	48.23%	81.25%	60%	40%	100%	25%
	Ertapenem (ETP)	52.32%	76.92%	71.42%	60%	66.66%	75%
	Imipenem (IMP)	46.06%	75%	62.5%	40%	100%	50%
Aminoglycosides	Amikacin (AK)	18.8%	57.14%	80%	40%	33.33%	25%
	Gentamicin (CN)	52.27%	56.33%	50%	60%	33.33%	25%
Quinolones	Ciprofloxacin (CIP)	77.64%	67.14%	50%	40%	0%	25%
Polymyxins	^**1**^ Colistin(CT)	11.36%	21.12%	0%	0%	0%	0%
	^**2**^ Polymyxin B(PB)	1.16%	13.84%	14.28%	20%	0%	0%
Sulfonamides/diaminopyrimidines	Sulfamethoxazole/Trimethoprim (SXT)	90.58%	90.76%	71.42%	100%	33.33%	100%
Tetracyclines Glycylclines	Tetracycline (TE)	80.89%	69.01%	33.33%	80%	33.33%	50%
	^**3**^ Tigecycline (TGC)	8.13%	28.12%	28.57%	40%	33.33%	100%
Phosphonic acidderivative	^**4**^ Fosfomycin (FF)	90.58%	98.38%	62.5%	100%	100%	100%
Rifamycins	Rifamycin (RA)	100%	93.65%	40%	100%	66.66%	100%

^**A**^ from 1 to 4 isolates of *E*.*coli*

^**B**^ from 1 to 9 isolates of *Klebsiella* spp.

^**C**^ from 1 to 3 isolates of *A*. *baumannii*were not tested by certain antibiotic disks and thus were excluded from percentage.

^**1**^ Zone diameter less than or equal to 10mm was interpreted as resistant according to CLSI, 2010

^**2**^ Zone diameter less than or equal to 11mm was interpreted as resistant according to CLSI, 2010

^**3**^ Zone diameter less than or equal to 15mm (for *E*.*coli* only) was interpreted as resistant according to EUCAST, 2018

^**4**^ Zone diameter less than or equal to 12mm (for *E*.*coli* causing urinary tract infections) was interpreted as resistant according to CLSI,2017

**Fig 1:**
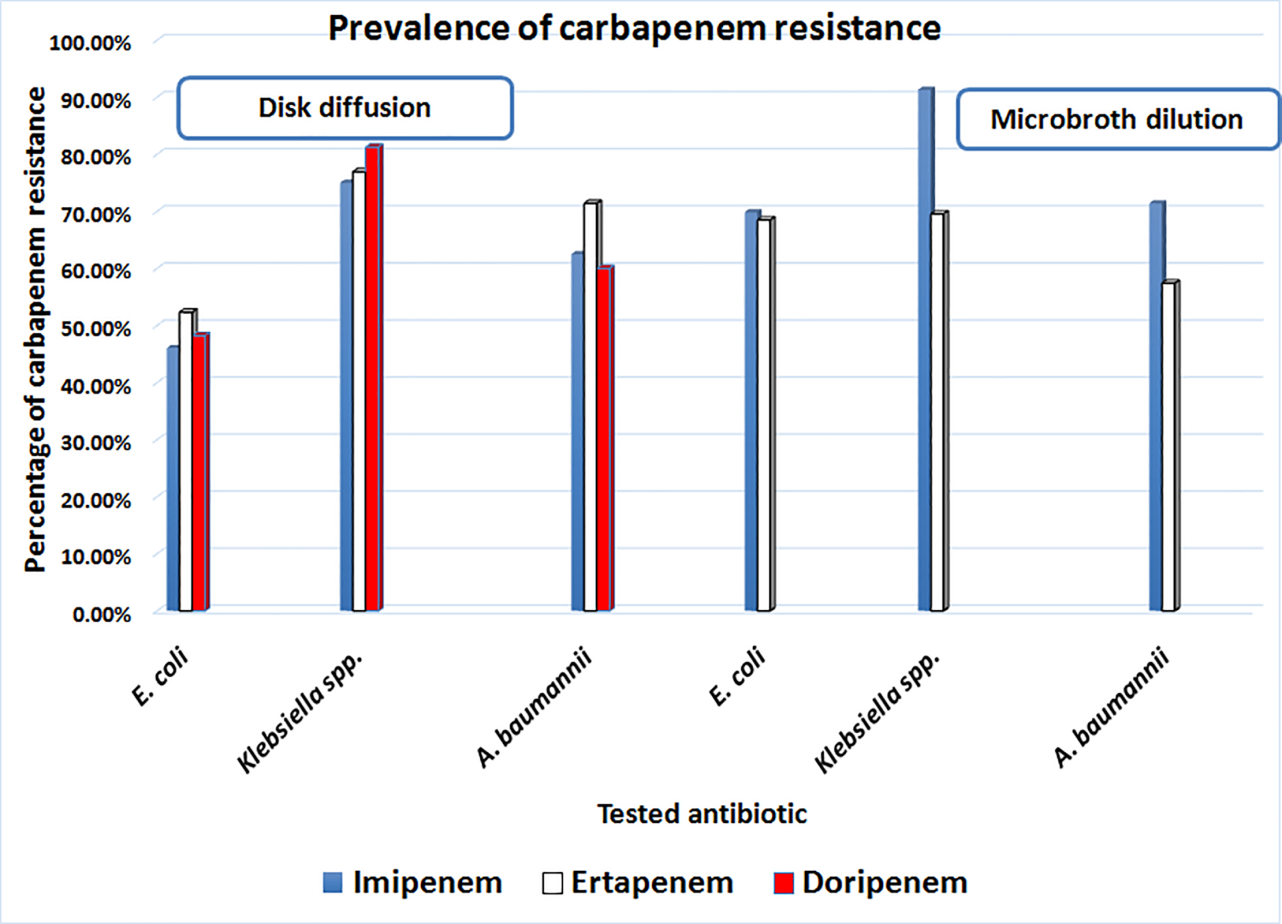
Prevalence of carbapenem resistant isolates.

Prevalence of carbapenem resistance and screening for potential CPs as determined by disk diffusion and MIC by broth microdilution method is summarized in Fig 1. The results of disk diffusion revealed that *Klebsiella* spp. showed high rate of resistance to doripenem (81%) followed by ertapenem (76%) and imipenem (75%).*A*. *baumannii* showed high resistance rate to ertapenem (71.4%) followed by imipenem (62.5%) and doripenem (60%). *E*. *coli* showed high resistance pattern to ertapenem (52.3%), followed by doripenem (48.2%) then imipenem (46%).The results of MIC by broth microdilution method reveals that out of 73 isolates of *E*. *coli* 50 (68.49%) and 51 (69.86%) were ertapenem and imipenem resistant, respectively. Out of 46 isolates of *Klebsiella* species 32 (69.56%) and 42 (91.30%) were ertapenem and imipenem resistant, respectively. Isolates showing MIC greater than or equal to 2 μg/ml for ertapenem and MIC greater than or equal to 4 μg/ml for imipenem were considered carbapenem resistant isolates with high potential for production of carbapenemase enzymes.

### Phenotypic detection of selected carbapenemase producers isolate by different methods

Isolates that showed reduced sensitivity by disk diffusion and MIC were selected for performing phenotypic tests. The ability of *E*. *coli* and *Klebsiella* spp. to produce carbapenemase enzyme was determined by using MHT, m CIM, blue carba test and combined disk test as shown in Table 3.

**Table 3:** Phenotypic detection of carbapenemase producers.

Tested isolate	Modified Hodge test (MHT)^1^				Modified carbapenem inactivation method (mCIM)^2^		Blue carba test^3^		Combined disk test^4^			
	Ertapenem disk		Meropenem disk		Meropenem disk		Imipenem powder		Meropenem/boronic acid (Class A)		Imipenem/EDTA (Class B)	
	No of cpo*/total no tested	%	No of cpo*/total no tested	%	No of cpo*/total no tested	%	No of cpo*/total no tested	%	No of cpo*/total no tested	%	No of cpo*/total no tested	%
***E*. *coli***	27/50	54	21/50	42	3/7	42%	6/7	85	2/11	18.8%	8/11	72.7
***Klebsiella spp*.**	15/40	37.5	13/40	32.5	4/8	50%	6/8	75	2/10	20%	7/10	70
***Ent*. *cloacace***	2/5	40	1/5	20	nd	-	nd	-	nd	-	nd	-
***A*. *baumannii***	1/4	25	0/4	0	nd	-	nd	-	nd	-	nd	-
***St*. *maltophilia***	0/3	0	0/3	0	nd	-	nd	-	nd	-	nd	-

^1^ Modified Hodge test was done in duplicate as an initial screening test for determination of carbapenemase-production among carbapenem-resistant isolates

^2^Modified carbapenem inactivation method was done in duplicate on promising CPs isolates after plasmid extraction

^3^Blue carba test was done in duplicate on the promising CPs isolates after plasmid extraction

^4^Combined disk test was done on CPs isolates in duplicate to confirm the presence of carbapenemase class A and class B.

*cpo = carbapenemase producing organismNd = not determined

### Plasmid extraction and PCR amplification of carbapenemase-producing isolates

Out of 32 isolates (12 *E*. *coli*, 13 *Klebsiella* spp, 3 *A*. *baumannii* and 1 of each *Proteus*, *Salmonella*, *St*. *maltophilia* and *Pseudomonas* spp.) were tested for plasmid mediated carbapenemase genes, 29 had showed faint plasmid bands. The results of PCR reveals that *bla*_oxa-48_ was observed in 17 (8 *E*. *coli*, 7 *Klebsiella* spp,1 from *A*. *baumannii* and *Salmonella* spp. Eight isolates were positive for *bla*_NDM_ (2 *E*. *coli*, 3 *Klebsiella* spp. 1 from each *A*. *baumannii*, *Proteus* spp. and *Pseudomonas* spp. Three isolates were positive for *bla*_VIM_ (2 *E*. *coli* and 1 *St*. *maltophilia*. Only 1 *E*. *coli* isolate was positive for both *bla*_NDM_ and *bla*_VIM_. Two isolates were positive for *bla*_KPC_ (1 *E*. *coli* and 1 *Klebsiella* spp.). No *bla*_IMP_ was carried on tested isolates.

## Discussion

Infections caused by MDR bacteria, especially CR GNB are becoming increasingly prevalent and constitute a real threat to public health that is associated with high morbidity and mortality rate among cancer patients. In the current study the majority of the recovered GN isolates were from blood culture(n = 146, 78.9%), followed by surgical specimens including pus, wound and swabs (n = 12, 6.4%) and others including catheter tip and sputum (n = 12, 6.4%).The predominance of blood culture among recovered specimen is a true reflection of blood stream infections with opportunistic bacterial and fungal pathogens among leukemic patients receiving aggressive and prolonged chemotherapy[24].Out of 171 enterobacterial isolates, 86 (50%) were *E*. *coli*, 71 (41.52%) were *Klebsiella* spp., 5 (2.9%) for each *Ent*. *cloa*caeand *Proteus* spp., 3 (1.7%) *Salmonella* spp. and 1 (0.5%) *S*. *marcescens*. Out of 14 non fermentive bacteria 7 (50%) *A*. *baumannii*, 4 (28.5%) *Pseudomonas* spp. and 3 (21.4%)*St*. *maltophilia*. The microbiological data of the current study was in accordance with other recent studies that emphasis on the constant threats of GNB especially *E*. *coli*, *Klebsiella* spp. and *Pseudomonas* spp. among leukemic host[25,26].It is a matter of concern that out of 171 enterobacterial isolates, 161 (94.15%) showed resistance to three or more antimicrobial class and were considered multidrug resistant. Of these, 84 out of 86 (97.6%) were for *E*. *coli*, 68 (95.77%) out of 71 were for *Klebsiella* spp. 3 out of 5 (60%) were for *Proteus* spp., 5 (100%) for *Ent*. *cloacae* and 1 (100%) for *Serratia marcescens*. Out of 14 non fermentive bacilli, 4 (100%) for *Pseudomonas* spp. and 3 (100%) for *St*. *maltophilia* and 5 out of 7 (71.42%) for *A*. *baumannii*. Although, the antibiogram analysis of recovered GNB is perturbing, still colistin, polymyxin B and tigecycline represent an array of hope by showing an overall sensitivity pattern ranging from 80% to 92%.

Moreover, the rate of carbapenem resistance displayed a worrisome trend as 113 out of 171 enterobacterial isolates (66.08%) and 12 out of 14 non fermentive bacilli (85.71%) showed resistant pattern to at least one of the tested carbapenem (ertapenem, meropenem and imipenem) by disk diffusion method. Owing to the massive spread of CP GNB with overwhelming consequences among health care settings, rapid detection is of vital importance for prompt implantation of infection control measures and selecting optimum antimicrobial therapy. Preliminary screening of carbapenemase producers was done using MHT (ertapenem and meropenem disk were used as substrate). Of the 90 enterobacterial isolates tested in the current study 42 (46.66%) with ertapenem disk and 34 (37.77%) with meropenem disk were MHT positive. Hence, ertapenem can beconsidered a more sensitive substrate for carbapenemase production. Recent studies conducted by Al Tamimi *et al*. and ElMahallawy *et al*., had reported (43%) and(49%), respectively with the MHT results [27–28]. In the current study, relatively low percentage of carbapenemase detection by MHT could be attributed to presence of class B carbapenemase including NDMs, VIMs and IMPs that requires zinc for their action[22]. To discriminate between the classes of carbapenemases, boronic acid and EDTA were used in addition to suitable substrate to inhibit class A and Class B, respectively in the combined disk test. Out of 21 isolates 4 (19.04%) and 15 (71.42%) were positive for class A and class B carbapenemase, respectively. This indicated that other carbapenemase type like class D oxacilinase (not inhibited by boronic acid or EDTA) might be involved in carbapenem-resistance pattern. In an attempt to cope with recent guidelines for carbapenemase production, m CIM and blue carba test were tested against 15 of the phenotypically confirmed CPs by previous methods. The results revealed that the former method detected 7 (46.6%), while the latter test detected 12 (80%) of CP enterobacterial isolates. Accordingly, blue carba test is considered a promising biochemical test for rapid determination of CPs within clinical settings.

After phenotypic screening of carbapenemase-producing isolates, we planned to detect the prevalence of plasmid mediated carbapenemase genes. Almost 90.62% of tested isolates showed plasmid bands upon running on 0.8% agarose gel. Thus, prompt detection is critical for the suppression of such nightmare strains within hospital settings. All the phenotypically confirmed plasmid mediated carbapenemase gene (29 isolates) were subjected to multiplex PCR using *bla*_KPC_, *bla*_IMP_, *bla*_VIM_, *bla*_NDM_ and *bla*_OXA-48_specific primers. The results revealed that *bla*_OXA-48_was the most prevalent 17 (58.62%), followed by *bla*_NDM_8(27.58%), then *bla*_VIM_3 (10.3%) and finally *bla*_KPC_2 (6.89%). Interestingly, one *E*. *coli* isolate harbored more than one type of metallo beta lactamase (*bla*_VIM_, *bla*_NDM_). Although, OXA-48 was initially identified in *K*. *pneumoniae* from Turkey, however since then OXA-48 producing enterobacterial strains has been widely spread as a source of nosocomial outbreaks in Mediterranean countries including Egypt as reported by many studies [27,29,30].This high level of OXA-48 among cancer patients is alarming due to difficulty in detection by reliable phenotypic methods, association with treatment failure and high rate of dissemination via transferable plasmids[31]. Also, the presence of NDM producers among our isolates should not be underestimated as they harbor multiple plasmid and chromosome mediated resistance genes, resulting in a MDR phenotype[32]. Finally, summarization of phenotypic and molecular characters of some CP isolates as shown in Table 4 revealed that MIC of three isolates no (2, 192 and 163)were not elevated this was attributed to production of *bla*_oxa-48_ that is associated with poor hydrolysis to carbapenem substrate. It was not surprising that combined disk test failed to detect two *bla*_oxa-48_ producing isolates however, temocillin is under investigation for inhibiting *bla*_oxa48_[33]. Concerning mCIM it showed negative results with three isolates producing *bla*_oxa48_and one isolate producing *bla*_KPC_. This could also be attributed to weak hydrolytic activity of *bla*_oxa48_ to meropenem as reported by other studies[34,35]. However, another study had reported positive results with *bla*_OXA-48_ producing isolates that were difficulty detected by phenotypic methods [36]. Interestingly, blue carba test was able to rapidly detect all variants of carbapenemase genes[17]. Based on the gigantic genetic diversity among the widely spread carbapenemase enzymes, it is absolutely necessary to perform molecular test in addition to detailed phenotypic screening test for better correlation of results. Although, the molecular methods can retain the inherit drawback of being coasty and unable to detect novel genes it is still the gold standard method [37].

**Table 4:** Summarization of phenotypic and genotypic characters of some carbapenemase- producing *E*. *coli* and *Klebsiella* spp.

Isolate no	isolate	MIC imipenemμg/ml	MIC ertapenemμg/ml	Modified hodge test	Combined disk test	Modified carbapenem inactivation	Blue carba test	Carbapenemase genes
2	*E*. *coli*	8	16	+ M + E	+A	+VE	+VE	*bla*_OXA-48_
192	*Klebsiella* spp.	8	4	+ M + E	-A -B	-VE	+VE	*bla*_OXA-48_
188	*Klebsiella* spp.	>256	>256	+E +M	+ A + B	-VE	+ VE	*bla*_OXA-48_
186	*Klebsiella* spp.	>256	>256	+M +E	-A -B	-VE	+VE	*bla*_KPC_
190	*Klebsiella* spp.	>256	>256	+M +E	+B	+VE	+VE	*bla*_NDM_
163	*E*. *coli*	4	2	+M +E	-A -B	-VE	+VE	*bla*_OXA-48_
181	*Klebsiella* spp.	>256	>256	-M +E	-A +B	+VE	+VE	*bla*_NDM_
150	*Klebsiella* spp.	>256	>256	+M +E	+B	+VE	+VE	*bla*_OXA-48_
167	*E*. *coli*	>256	>256	+E -M	+B	+VE	+VE	*bla*_NDM_
183	*Klebsiella* spp.	>256	>256	+M +E	+B	+VE	+VE	*bla*_NDM_

Abbreviations: M = meropenem, E = ertapenem, A = class A, B = class B

## Conclusion

The antibiogram analysis of GNB recovered from children with malignancy showed a remarkable high resistant pattern towards most of the tested antibiotic particularly β-lactam group including, monobactam, amoxicillin/clavulanic acid, 3^rd^and 4^th^ generation cephalosporins and carbapenems, fosfomycin and rifamycin. The prevalence of enterobacterial isolates harboring *bla*_OXA-48_or *bla*_NDM_ is a rising threat in Egypt that requires immediate application of active antimicrobial stewardship programs that deescalate the use of mainstay carbapenem and polymyxins.
